# Functional classification of RUNX1 variants in familial platelet disorder with associated myeloid malignancies

**DOI:** 10.1038/s41375-021-01200-w

**Published:** 2021-03-10

**Authors:** Melanie Decker, Tim Lammens, Alina Ferster, Miriam Erlacher, Ayami Yoshimi, Charlotte M. Niemeyer, Martijn P. T. Ernst, Marc H. G. P. Raaijmakers, Nicolas Duployez, Andreas Flaum, Doris Steinemann, Brigitte Schlegelberger, Thomas Illig, Tim Ripperger

**Affiliations:** 1 Department of Human Genetics, Hannover Medical School, Hannover, Germany; 2 Department of Pediatric Hematology-Oncology and Stem Cell Transplantation, Ghent University Hospital, Ghent, Belgium; 3 Hematology-Oncology, Queen Fabiola Children’s University Hospital (ULB), Brussels, Belgium; 4 Division of Pediatric Hematology-Oncology, Department of Pediatric and Adolescent Medicine, University of Freiburg, Freiburg, Germany; 5 Department of Hematology, Erasmus MC Cancer Institute, Rotterdam, The Netherlands; 6 Department of Hematology, CHU Lille, INSERM, University Lille, Lille, France; 7 Hannover Unified Biobank, Hannover Medical School, Hannover, Germany

**Keywords:** Cancer genetics, Genetic testing, Translational research, Cancer genetics, Genetics research

## To the Editor:

Heterozygous, pathogenic germline variants of *RUNX1* [1] are causative for familial platelet disorder with associated myeloid malignancies (RUNX1-FPD, FPDMM, FPD/AML; OMIM 601399; ORPHA 71290) [2]. RUNX1-FPD is characterized by incomplete penetrance and a broad spectrum of clinical phenotypes, even within affected families [3, 4]. The clinical presentation includes thrombocytopenia, most frequently moderate, functional platelet defects, and a risk of ~44% [5] to develop a hematological malignancy, mainly myelodysplastic syndrome and acute myeloid leukemia, but rarely also T-cell acute leukemia [6]. Many *RUNX1* variants are reported only in individual families [3], hence they have not been associated with RUNX1-FPD before and no functional data is available. In silico prediction tools (e.g., rare exome variant ensemble learner (REVEL) [7]) are frequently not convincing, especially for variants in the highly conserved and frequently affected Runt homology domain (RHD) (Supplementary Fig. 1). According to present classification rules [5], *RUNX1* variants must be frequently classified as variants of uncertain significance (VUS). Lately, the ClinGen Myeloid Malignancy Variant Curation Expert Panel (MM-VCEP) has published adjusted ACMG/AMP guidelines for *RUNX1* including recommendations how to integrate functional data in variant classification [5].

**Fig. 1:**
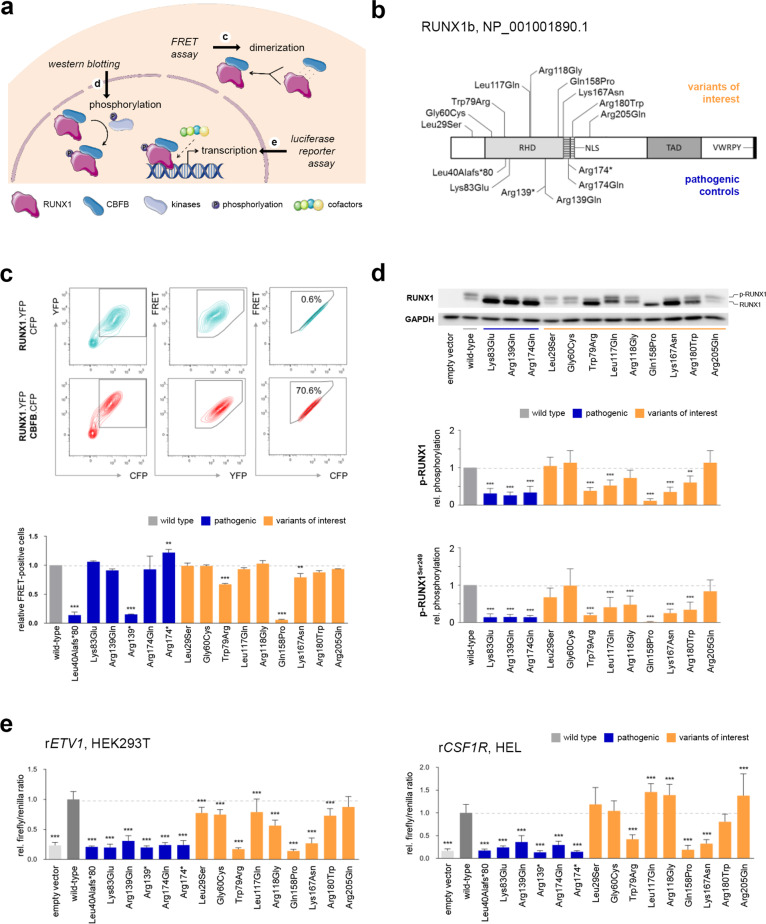
Functionally addressed processes, variants investigated, and representative results. **a** The ability of RUNX1 variants to form heterodimers with CBFB was analyzed using a flow-cytometry-based FRET assay. Phosphorylation of RUNX1 missense variants was quantified by means of western blotting. Transcriptional activation through RUNX1 variants was investigated using luciferase reporter assays. This figure was created using free, adapted images from Servier Medical Art, licensed under a Creative Common Attribution 3.0 Generic License. http://smart.servier.com. **b** Schematic overview of the investigated RUNX1 variant set and relevant RUNX1 domains based on Lam and Zhang [1]. The variant nomenclature refers to RUNX1 transcript variant 2 (NM_001001890.3), which encodes for isoform b of RUNX1 (453 amino acids). For variant denotations with respect to RUNX1 isoform c and additional information please refer to Supplementary Data. **c** Representative contour plots of HEK293T cells after cotransfection of RUNX1.YFP with CFP (upper panel) and CBFB.CFP (lower panel) indicating from left to right the gating strategy: (1) gating of YFP^+^CFP^+^-positive cells, (2) exclusion of false positive FRET^+^ signals resulting from YFP excitation by the 405 nm laser, and (3) detection of FRET^+^ signals after adjusting the gate following cotransfection of RUNX1.YFP with CFP that should be FRET^–^. Below the dot plot panels, the bar graph displays the amount of FRET^+^ cells relative to wild-type RUNX1 (mean + standard deviation (SD); three biological replicates; one-way ANOVA; Dunnett’s post hoc test; *P ≤ 0.05; **P ≤ 0.01; ***P ≤ 0.001). **d** Representative western blot result. As indicated, the upper and lower RUNX1 band represents the phosphorylated and unphosphorylated form of RUNX1, respectively. The lower panel displays GAPDH which was analyzed as loading control. The bar graph shows the quantification of the amount of phosphorylated and Ser249-phosphorylated RUNX1 protein relative to wild-type RUNX1 (mean + SD; three biological and two technical replicates; one-way ANOVA; Dunnett’s post hoc test; *P ≤ 0.05; **P ≤ 0.01; ***P ≤ 0.001). **e** The left and right bar graph displays the firefly/renilla ratios relative to wild-type RUNX1 for the reporter constructs r*ETV1* in HEK293T (mean + SD; three biological and three technical replicates) and r*CSF1R* in HEL (mean + SD; two biological and five technical replicates), respectively (one-way ANOVA; Dunnett’s post hoc test; *P ≤ 0.05; **P ≤ 0.01; ***P ≤ 0.001).

To functionally characterize nine previously reported *RUNX1* variants and complement the recent MM-VCEP guidelines [5], we developed a set of functional assays addressing heterodimerization with CBFB, phosphorylation of RUNX1, and the ability of RUNX1 to activate transcription. For controls, 6 known pathogenic variants and wild-type RUNX1 were investigated in parallel (Fig. 1a, b). We used a scoring system to ascertain the functional class of investigated variants and determine their clinical classification by applying the MM-VCEP guidelines integrating our functional data. For detailed information about variants investigated (including HGVS nomenclature regarding RUNX1b [NM_001001890.3] and its transfer to RUNX1c [NM_001754.5], genomic localization (GRCh38), ClinVar and dbSNP links, REVEL score, and available clinical information), materials, and methods please refer to Supplementary Data.

The interaction of RUNX1 and its cofactor CBFB is essential for efficient and stable DNA binding of the resulting transcription factor complex. To determine the heterodimerization ability of RUNX1 variants, we performed a FRET assay using YFP and CFP fusion proteins in HEK293T cells (Fig. 1c, Supplementary Fig. 2). In comparison with the wild-type protein, two pathogenic RUNX1 variants (i.e., Leu40Alafs*80 and Arg139*) and the variant Gln158Pro failed to efficiently dimerize with CBFB. Variants Trp79Arg and Lys167Asn showed moderately reduced heterodimerization, whereas all other variants showed heterodimerization to at least 85% of wild-type activity.

Phosphorylation influences the ability of RUNX1 to activate transcription and affects its stability [8]. We coexpressed RUNX1 or its variants with CBFB in HEK293T cells and quantified the proportion of phosphorylated RUNX1. Additionally, Ser249-phosphorylation of RUNX1 was investigated (Fig. 1d, Supplementary Fig. 3A). Our data revealed reduced phosphorylation for all pathogenic missense controls (i.e., Lys83Glu, Arg139Gln, and Arg174Gln) and for six out of nine variants of interest (i.e., Trp79Arg, Leu117Gln, Arg118Gly, Gln158Pro, Lys167Asn, and Arg180Trp).

Since RUNX1 CBFB complexes function as transcriptional activators, we used four independent luciferase reporters to examine the ability of RUNX1 variants to activate transcription in human nephrogenic HEK293T cells (i.e., r*CSF1R*, r*ETV1*, r*MYL9*, and r*PDE4DIP*). We observed omitted transcriptional activation for all pathogenic RUNX1 variants and for three out of nine variants of interest (i.e., Trp79Arg, Gln158Pro, and Lys167Asn). The variants Gly60Cys and Arg205Gln mainly resembled the activity of wild-type RUNX1, whereas the remaining four variants (i.e., Leu29Ser, Leu117Gln, Arg118Gln, and Arg180Trp) showed varying levels of impaired transcriptional activation (Fig. 1e, Supplementary Fig. 4).

To further evaluate the transcriptional activation ability of RUNX1 variants in a hematopoietic context, we additionally performed luciferase assays in the human erythroid leukemia cell line (HEL) using two independent reporter constructs (i.e., r*ETV1* and r*CSF1R*, Fig. 1e, Supplementary Figs. 5, 6). In line with the results for nephrogenic HEK293T cells, we detected substantially decreased transcriptional activation for all pathogenic controls and for three out of nine variants of interest (i.e., Trp79Arg, Gln158Pro, and Lys167Asn).

**Fig. 2:**
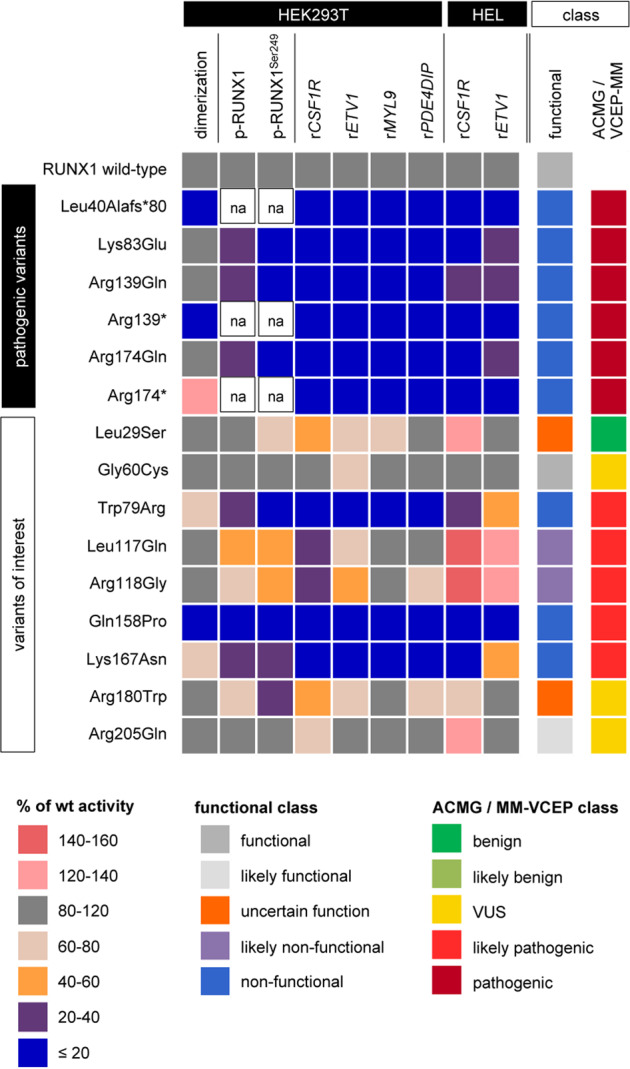
Variant scoring and classification. For all assays, the heatmap summarizes the activity of each variant relative to wild-type RUNX1 in HEK293T and HEL assays. In addition, the functional and ACMG/MM-VCEP classes [5] are given. Color codes are shown below. wt wild type, na not applicable, VUS variant of uncertain significance.

After developing and applying individual functional assays comparable to previous investigations [9], we applied a scoring system to integrate results of individual assays for each RUNX1 variant (Fig. 2). Based on the recommendations of the MM-VCEP [5] and our functional data, we classified variants with ≥2 scores <20% as *non-functional* (PS3_strong), variants with ≥3 scores <60% or >140% as *likely non-functional* (PS3_moderate), variants with ≤1 scores <80% or >120% as *functional* (BS3_strong), variants with ≤2 scores <80% or >120% as *likely functional* (BS3_supporting), and variants with conflicting results not allowing final conclusions as *uncertain*. We identified three out of nine variants (i.e., Trp79Arg, Gln158Pro, and Lys167Asn) resembling the loss-of-function defect seen for known pathogenic variants and classified them as *non-functional*. For two additional variants (i.e., Leu117Gln and Arg118Gly) classified as *likely non-functional*, there was also clear evidence for their functional impairment; however to a lesser extent than for the variants Trp79Arg, Gln158Pro, and Lys167Asn. In contrast, variants Gly60Cys and Arg205Gln displayed functional activity comparable to wild-type RUNX1 and were classified as *functional* and *likely functional*, respectively. Functionally, no conclusive results were obtained for Leu29Ser and Arg180Trp, which we thus called functionally *uncertain*.

As a proof of principle, we show that our assays confirm the non-functionality of all six pathogenic controls investigated, which include missense, nonsense, and frameshift variants. Based on our results, out of nine investigated variants, three variants were classified as *non-functional* (i.e., Trp79Arg, Gln158Pro, and Lys167Asn), two variants as *likely non-functional* (i.e., Leu117Gln and Arg118Gly), and two variants as *likely functional* (i.e., Gly60Cys and Arg205Gln). Combining our experimental data and the MM-VCEP guidelines [5] (Fig. 2, Supplementary Data 2C), we classified Trp79Arg, Leu117Gln, Arg118Gly, Gln158Pro, and Lys167Asn as likely pathogenic. Our results of Trp79Arg confirmed previous functional data [10] and reclassification by the MM-VCEP [5].

The analysis of the *functional* and *likely functional* variants Gly60Cys and Arg205Gln illustrates, that even in the presence of experimental data indicating functionality, rare variants cannot be classified as (likely) benign following the present expert recommendations. This is especially true if in silico tools (e.g., REVEL) predict variants to be deleterious, which is the case for the majority of variants located within the heavily conserved RHD (Supplementary Fig. 1). This aspect needs to be addressed in any future revision of the expert guidelines.

In line with previous investigations, our functional results regarding Leu29Ser allowed no final conclusion [11–13]. However, there is no doubt of the recent benign classification of Leu29Ser regarding its population frequency and the detection of homozygous carriers (e.g., gnomAD). The results nicely illustrates the MM-VCEP recommendation [5] that proper variant classification needs to carefully consider allele frequencies, segregation data, phenotypic criteria, and functional data in parallel.

In summary, our current set of assays detects all investigated known (likely) pathogenic variants in RUNX1. In combination with the recently published expert recommendations [5], assays designed and applied herein allow reclassification of four out of seven VUS as likely pathogenic. The three remaining variants keep their status as VUS, however, two of them are *(likely) functional* applying the present assays, but due to their REVEL score and low population frequency, they cannot be categorized as likely benign.

Reclassification of VUS has significant impact on patient care by guiding treatment decision, donor selection among matched relatives, and predictive testing in relatives. By addressing heterodimerization, phosphorylation, and transactivation, we show that transactivation assays have—in line with the hierarchy of molecular events regarding RUNX1 function—the biggest influence on functional classification. Comparing data of reporter assays in nephrogenic HEK293T and hematopoietic HEL cells, HEK293T-based assays provide reliable functional results. Thus, based on our data and following the expert guidelines [5], we recommend reporter assays in easy-to-transfect HEK293T for first-line screenings of variants of interest, followed by additional assays if necessary to apply the PS3 criterion.

Finally, there is a constantly increasing need for functional assays due to the vast application of next-generation sequencing. Consequently, the development of high-throughput assays is required to enable fast and accurate functional classification of thousands of variants in parallel [14]. Set up and application of these high-throughput tools considering recent recommendations [15] will generate a set of functional data of many RUNX1 variants, even before their first genetic report, and can thus support classification of the majority of novel variants. Based on such functional data, future revisions of the MM-VCEP RUNX1 guidelines can define application rules and thresholds for strong, moderate, and supporting functional evidence considering results of benign and pathogenic controls.

## Supplementary information

Supplemental data

## References

[1] Lam K, Zhang D-E (2012). RUNX1 and RUNX1-ETO: roles in hematopoiesis and leukemogenesis. Front Biosci..

[2] Song WJ, Sullivan MG, Legare RD, Hutchings S, Tan X, Kufrin D (1999). Haploinsufficiency of CBFA2 causes familial thrombocytopenia with propensity to develop acute myelogenous leukaemia. Nat Genet..

[3] Brown AL, Arts P, Carmichael CL, Babic M, Dobbins J, Chong CE (2020). RUNX1-mutated families show phenotype heterogeneity and a somatic mutation profile unique to germline predisposed AML. Blood Adv..

[4] Brown AL, Hahn CN, Scott HS (2020). Secondary leukemia in patients with germline transcription factor mutations (RUNX1, GATA2, CEBPA). Blood..

[5] Luo X, Feurstein S, Mohan S, Porter CC, Jackson SA, Keel S (2019). ClinGen myeloid malignancy variant curation expert panel recommendations for germline RUNX1 variants. Blood Adv..

[6] Schlegelberger B, Heller PG (2017). RUNX1 deficiency (familial platelet disorder with predisposition to myeloid leukemia, FPDMM). Semin Hematol..

[7] Ioannidis NM, Rothstein JH, Pejaver V, Middha S, McDonnell SK, Baheti S (2016). REVEL: an ensemble method for predicting the pathogenicity of rare missense variants. Am J Hum Genet.

[8] Imai Y, Kurokawa M, Yamaguchi Y, Izutsu K, Nitta E, Mitani K (2004). The corepressor mSin3A regulates phosphorylation-induced activation, intranuclear location, and stability of AML1. Mol Cell Biol..

[9] Michaud J, Wu F, Osato M, Cottles GM, Yanagida M, Asou N (2002). In vitro analyses of known and novel RUNX1/AML1 mutations in dominant familial platelet disorder with predisposition to acute myelogenous leukemia: Implications for mechanisms of pathogenesis. Blood..

[10] Tsai SC, Shih LY, Liang ST, Huang YJ, Kuo MC, Huang CF (2015). Biological activities of RUNX1 mutants predict secondary acute leukemia transformation from chronic myelomonocytic leukemia and myelodysplastic syndromes. Clin Cancer Res..

[11] Huang G, Zhao X, Wang L, Elf S, Xu H, Zhao X (2011). The ability of MLL to bind RUNX1 and methylate H3K4 at PU.1 regulatory regions is impaired by MDS/AML-associated RUNX1/AML1 mutations. Blood..

[12] Koh CP, Wang CQ, Ng CEL, Ito Y, Araki M, Tergaonkar V (2013). RUNX1 meets MLL: epigenetic regulation of hematopoiesis by two leukemia genes. Leukemia..

[13] Duployez N, Fenwarth L (2020). Controversies about germline RUNX1 missense variants. Leuk Lymphoma..

[14] Findlay GM, Daza RM, Martin B, Zhang MD, Leith AP, Gasperini M (2018). Accurate classification of BRCA1 variants with saturation genome editing. Nature..

[15] Brnich SE, Abou Tayoun AN, Couch FJ, Cutting GR, Greenblatt MS, Heinen CD (2020). Recommendations for application of the functional evidence PS3/BS3 criterion using the ACMG/AMP sequence variant interpretation framework On behalf of the Clinical Genome Resource Sequence Variant Interpretation Working Group. Genome Med..

