# Correction to: Functional classification of RUNX1 variants in familial platelet disorder with associated myeloid malignancies

**DOI:** 10.1038/s41375-022-01709-8

**Published:** 2022-10-18

**Authors:** Melanie Decker, Tim Lammens, Alina Ferster, Miriam Erlacher, Ayami Yoshimi, Charlotte M. Niemeyer, Martijn P. T. Ernst, Marc H. G. P. Raaijmakers, Nicolas Duployez, Andreas Flaum, Doris Steinemann, Brigitte Schlegelberger, Thomas Illig, Tim Ripperger

**Affiliations:** 1 Department of Human Genetics, Hannover Medical School, Hannover, Germany; 2 Department of Pediatric Hematology-Oncology and Stem Cell Transplantation, Ghent University Hospital, Ghent, Belgium; 3 Hematology-Oncology, Queen Fabiola Children’s University Hospital (ULB), Brussels, Belgium; 4 Division of Pediatric Hematology-Oncology, Department of Pediatric and Adolescent Medicine, University of Freiburg, Freiburg, Germany; 5 Department of Hematology, Erasmus MC Cancer Institute, Rotterdam, The Netherlands; 6 Department of Hematology, CHU Lille, INSERM, University Lille, Lille, France; 7 Hannover Unified Biobank, Hannover Medical School, Hannover, Germany

Correction to: *Leukemia* 10.1038/s41375-021-01200-w, published online 10 March 2021

In the original article, all Lys167Arg-mislabelled variants were changed to Lys167Asn. Furthermore, the Supplementary Information was revised.

The original article has been corrected.

