# Supplementary material for: Functional classification of RUNX1 variants in familial platelet disorder with associated myeloid malignancies
Source: Leukemia. 2021 Mar 10;35(11):3304–8. doi: 10.1038/s41375-021-01200-w (PMC8550979; doi:10.1038/s41375-021-01200-w)
Supplement: Supplementary file 1 — Supplemental data [file 41375_2021_1200_MOESM1_ESM.docx]

**Supplemental Data**

**1. Experimental procedures**

**2. Investigated RUNX1 variants**

**A** pathogenic controls

**B** variants of interest

**C** classification of variants of interest

**3. Supplementary Figures and Table**

**1. Experimental procedures**

**Cloning of cDNA expression and luciferase reporter plasmids**

Coding sequences of *RUNX1* transcript variant 2 (NM_001001890.3) and *CBFB* (NM_001755.3) were PCR-amplified using primers tagged with restriction sites. Upstream of the start codon, the Kozak element (i.e., 5’-GCCACC-3’) was introduced. Tagged restriction sites were used for directed ligation into pcDNA3.1/Zeo(+) (Thermo Fisher). Subsequently, RUNX1 variants were introduced by site-directed mutagenesis.

For CFP and YFP fusion proteins, coding sequences of wild-type *RUNX1*, its variants, and *CBFB* were amplified by PCR and subcloned into pEYFP-C1 and pECFP-C1 (kindly provided by Michael Schindler; HZI, Munich, Germany) following the previously described cloning strategy [1].

For luciferase reporter constructs, pGL3.basic (Promega) was used. Promotor region fragments of *ETV1, PDE4DIP, MYL9,* and *CSF1R* obtained by PCR amplification of genomic DNA were subcloned upstream of its firefly luciferase expression cassette. The constructs r*ETV1* and r*PDE4DIP* were generated by integrating the genomic fragment of 1000 bp immediately upstream of the start codon. For reporters r*MYL9* and r*CSF1R*, we subcloned the promoter fragment as previously described by Jalagadugula et al. [2] and Zhang et al. [3], respectively.

To generate pGL4.7TK[hRluc], the *Bgl*II and *Hind*III-flanked TK promoter fragment of pRL.TK (Promega) was inserted into pGL4.70[hRLuc] (Promega).

Plasmid DNAs were isolated using NucleoBond Xtra Midi Kits (Macherey-Nagel). However, for nucleofection (see below), plasmid DNAs were isolated using EndoFree Plasmid Maxi Kits (Qiagen). All generated vectors and vector inserts were verified by restriction digestion and Sanger sequencing, respectively. For primer sequences please refer to Supplementary Table 1.

**Flow cytometry-based FRET assay**

Cotransfection of YFP and CFP fusion protein expression plasmids into HEK293T cells was performed using the calcium phosphate method following standard procedures. One day prior to transfection, cells were seeded in DMEM without antibiotics in 6-well plates with a density of 4 x 10^5^ cells per well. Medium was replaced immediately before transfection by freshly prepared DMEM without antibiotics supplemented with 20 mM HEPES and 25 µM chloroquine. Per well and for each plasmid, 2.5 µg of plasmid DNA was transfected. Five to 6 hours after transfection, medium was replaced with fresh DMEM supplemented with 20 mM HEPES. Cells were harvested 24 h after transfection. They were washed twice with PBS (37°C), detached with PBS (37°C) by pipetting, and pelleted at 350 x g for 5 minutes at 4°C. Pellets were resuspended in ice-cold PBS supplemented with 4% FBS. Analyses were performed using an LSR II flow cytometer (CFP: violet laser (405 nm), 450/50 filter; YFP: blue laser (488 nm), 530/30 filter; FRET: violet laser (405 nm), 525/50 filter). Data was analyzed using FlowJo 7.2. We acquired 45 x 10^3^ YFP^+^-CFP^+^ events excluding false-positive events resulting from YFP excitation at 405 nm as described before [1]. For each RUNX1 construct the percentage of FRET^+^ cells was defined as the difference of YFP^+^-CFP^+^-FRET^+^ events in the independent cotransfections of the respective RUNX1 construct with pCBFB.ECFP-C1 or pECFP-C1. Gates were adjusted for wild-type RUNX1 and subsequently applied for the analyses of all variants. The percentage of FRET^+^ cells was plotted in comparison with wild-type RUNX1.

**Western blotting**

Protein lysates of HEK293T cells were obtained 24 h after transfection using RIPA lysis buffer. Proteins were separated by SDS-PAGE (5% stacking and 12% separation gel, 3 hours, 90 V_const_, room temperature) and transferred to PVDF membranes by tank blotting (ie 1 hour, 350 mA_const_, 4°C). Blocking was performed for 1 hour with 5% (w/v) milk powder or 5% (w/v) bovine serum albumin (for pRUNX1Ser249) in TBS supplemented with 1% Tween 20 at room temperature. The membranes were incubated with specific antibodies (RUNX1 1:5,000 (Cell Signaling, CST#4334); GAPDH 1:7,500 (Santa Cruz, sc25778); pRUNX1Ser249 1:1,000 (Cell Signaling, CST#4327) CBFB 1:20000(Cell Signaling CST#62184) overnight at 4°C and with the secondary goat anti-rabbit IgG-HRP antibody (Abcam, ab6721, 1:10,000 for RUNX1 and GAPDH, 1:5,000 for pRUNX1Ser249, 1:40000 for CBFB) for 2 hours at 4°C. Detection was performed with SuperSignal West Chemiluminescent Substrate. Intensities of RUNX1 signals were determined using Image Studio Lite Version 5.2, normalized to GAPDH, and analyzed relative to wild-type RUNX1.

**Luciferase reporter assays**

Using white 96-well plates, 8 x 10^3^ HEK293T cells were seeded per well in DMEM without antibiotics. After 24 hours, cells were cotransfected with 25 ng of a luciferase reporter plasmid, 2.5 ng pGL4.70[hRluc] renilla normalization vector (Promega), 100 ng pcDNA3.1.CBFB, and 100 ng pcDNA3.1.RUNX1 (ie wild-type or variant) using Lipofectamine 2000. Twenty-four h after transfection, firefly and renilla luciferase activity was measured using Dual-Glo Luciferase Assay System. Firefly/renilla ratios were analyzed relative to wild-type RUNX1.Transfection of HEL cells was performed by nucleofection using Cell Line Nucleofector Kit V (Lonza). Per cuvette, 4 x 10^6^ cells were transfected using Amaxa Nucleofector IIb program V-001. For each transfection, we combined 1 µg luciferase reporter plasmid, 50 ng pGL4.7TK[hRluc] renilla normalization vector, 2 µg pcDNA3.1.CBFB, and 2 µg pcDNA3.1.RUNX1 (ie wild-type or variant). Cells were harvested and counted 24 hours after transfection. Subsequently, 0.25 x 10^6^ cells were seeded per well of a 96-well plate and firefly and renilla luciferase activity were directly measured as described above.

**Statistics**

Data are presented as means ± standard deviation (SD). Data plotting and statistical analyses were performed using Prism 8, and statistically significant differences were determined by one-way of analysis of variance (ANOVA) with Dunnett’s post hoc test. Outliers were excluded to reach normal distribution, if necessary. *P*-values ≤.05 were considered as statistically significant (*, ≤.05; **, ≤.01; ***, ≤.001). FRET analyses were performed in biological triplicates. Quantification of phosphorylation by western blotting was performed in 3 biological and 2 technical replicates. Luciferase reporter assays in HEK293T cells were analyzed in 3 biological and 3 technical replicates. Luciferase reporter assays in HEL cells were analyzed in 2 biological and 5 technical replicates.

**Cell culture** Cells were grown at 37°C in a humidified atmosphere with 5% CO_2_. If not specified, DMEM (4.5 g/l glucose, 10% fetal bovine serum (FBS), 1 mM sodium pyruvate, and 100 U/ml penicillin/streptomycin) and RPMI 1640 (10% FBS, 2 nM L-glutamine, and 100 U/ml penicillin/streptomycin) were used to culture HEK293T and HEL cells, respectively.

**2. Investigated RUNX1 variants**

**A pathogenic controls**

| **RUNX1b (NM_001001890.3)** | **RUNX1c (NM_001754.5)** | **Chr21 (GRCh38)** | **ClinGen Allele Id** | **ClinVar RCV Id** | **dbSNP** | **REVEL** | **clinical information** |
| --- | --- | --- | --- | --- | --- | --- | --- |
| c.90_117dup p.(Leu40Alafs*80) | c.171_198dup p.(Leu67Alafs*80) | g.34886996_34887023dup | – | – | – | – | The index patient had asymptomatic thrombocytopenia (90 x 10^9^/l) and impaired *in vitro* platelet aggregation since his neonatal period. Asymptomatic thrombocytopenia was also known in his younger sister, his mother and his maternal grandfather. A maternal uncle had died from childhood leukemia. At age 5, the index patient was diagnosed with MDS-EB with normal karyotype. By Sanger sequencing, this heterozygous duplication inducing frame shift and premature truncation of RUNX1 was identified. The variant was also identified in his mother. The index patient underwent HSCT from a MUD. Two years after his diagnosis, a 17-year-old maternal uncle was also diagnosed with MDS-EB with normal karyotype. Successively, he progressed to AML with an unbalanced translocation, der(7)t(2;7)(p12;q21), and later on, a clonal evolution with trisomy 12. He also carried the familial *RUNX1* variant. Finally, the variant was also identified in the younger sister and an additional maternal uncle of the index patient. Both showed mild, asymptomatic thrombocytopenia without evidence of malignant transformation. |
| c.247A>G p.(Lys83Arg) | c.328A>G p.(Lys110Glu) | g.34886866T>C | CA248613 | RCV000680403.1  RCV000015551.27 | rs121912498 | 0.953 | The missense variant was included based on the report by Michaud et al. [4] describing a prototypic RUNX1-FPD family with thrombocytopenia, platelet aggregation defects, and leukemia in four generations. |
| c.416G>A p.(Arg139Gln) | c.497G>A p.(Arg166Gln) | g.34880568C>T | CA16616941 | RCV000477937.2 | rs1060499616 | 0.962 | This variant was reported in one of the original FPD-RUNX1 families reported by Song et al. [5]. The family history was characterized by thrombocytopenia and functional platelet defects. One of the affected individuals developed ‘preleukemia with trisomy 8’. |
| c.415C>T p.(Arg139*) | c.496C>T p.(Arg166*) | g.34880569G>A | CA10014502 | – | rs759068561 | – | The male index patient of this family had congenital, asymptomatic thrombocytopenia (60-80 x 10^9^/l). At age 3.5, platelet count declined over several months reaching values of 3-20 x 10^9^/l. Diagnostic bone marrow examination showed MDS-EB with normal karyotype. Thrombocytopenia was also known in his mother. The heterozygous truncating RUNX1 variant was identified in the index and his mother. The index patient underwent MUD HSCT. |
| c.521G>A p.(Arg174Gln) | c.602G>A p.(Arg201Gln) | g.34859485C>T | CA248610 | RCV000680425.1 RCV000015550.26 | rs74315450 | 0.94 | This variant was reported in the original study by Song et al. 1999 in a family with an autosomal dominant pattern of inheritance of thrombocytopenia and progression to AML [5]. |
| c.520C>T p.(Arg174*) | c.601C>T p.(Arg201*) | g.34859486G>A | CA16602487 | RCV000824700.1 | rs1057519748 | – | We previously reported this variant in a patient with childhood onset of MDR-AML with del(5q) and an unbalanced translocation der(2)t(2;6)(q36;q23). Her father also carrying the *RUNX1* variant had developed MDR-AML at age 47 [6]. |

**B variants of interest**

| **RUNX1b (NM_001001890.3)** | **RUNX1c (NM_001754.5)** | **Chr21 (GRCh38)** | **ClinGen Allele Id** | **ClinVar RCV Id** | **dbSNP** | **REVEL** | **clinical information** |
| --- | --- | --- | --- | --- | --- | --- | --- |
| c.86T>C p.(Leu29Ser) ^#^ | c.167T>C p.(Leu56Ser) | g.34887027A>G | CA10014578 | RCV000680395.1  RCV000226755.5  RCV000245382.1 | rs111527738 | 0.307 | This variant has previously been observed and reported several times in families with suspected RUNX1-FPD; and we included it to see its functional impact in our assays. Based on frequencies observed in population databases including homozygous carriers, it has become clear that this variant has no disease association and it has been classified as benign by the MM-VCEP [7]. |
| c.178G>T p.(Gly60Cys) | c.259G>T p.(Gly87Cys) | g.34886935C>A | CA10014559 | RCV000680398.1  RCV000527760.2  RCV000503460.1 | rs561166961 | 0.918 | While screening for HSCT donors, this missense variant was identified as germline variant in healthy relatives (i.e., sister and mother) of an index patient with Leu117Gln (please see below) suspected to have RUNX1-FPD. Blood counts were normal and there was no family history of bleeding disorder or hematological malignancies in the mother of the index patient. In the absence of other possible donors, responsible clinicians had to transplant the index with hematopoietic stem cells of the sister carrying this missense *RUNX1* VUS, even though poor donor cell engraftment and donor cell leukemia had previously been reported [8]. The transplant went fine and, up to now, follow-up was inconspicuous. |
| c.235T>A p.(Trp79Arg)^#^ | c.316T>A p.(Trp106Arg) | g.34886878A>T | CA410203508 | RCV000502317.1  RCV000824706.1 | – | 0.976 | The variant was reported as a variant of uncertain significance in ClinVar in an individual with suspected RUNX1-FPD. The variant was meanwhile classified as likely pathogenic by the MM-VCEP [7]. |
| c.350T>A p.(Leu117Gln) | c.431A>C p.(Leu144Gln) | g.34880634A>T | – | – | – | 0.985 | This missense variant was identified in a child with MDS (i.e., RAEB) diagnosed at five years of age after an episode of fever with thrombocytopenia, mild leucopenia and skin nodules. Cytogenetic studies showed an aberrant clone with 46,XY,+1,der(1;18)(q10;q10). RUNX1-FPD was suspected due the low platelet counts and aggregation-deficient platelets in his father having no bleeding history. The index patient required HSCT and received cells from his sister carrying Gly60Cys (please see above). Transplant went fine and, up to now, follow-up was inconspicuous. |
| c.352A>G p.(Arg118Gly) | c.433A>G p.(Arg145Gly) | g.34880632T>C | CA16616277 | RCV000472326.1 | rs757288001 | 0.925 | This variant was included based on its report as a VUS in ClinVar in an individual with suspected RUNX1-FPD. No additional information is available. |
| c.473A>C p.(Gln158Pro) | c.554A>C p.(Gln185Pro) | g.34859533T>G | CA410208062 | RCV000680420.1 | – | 0.977 | The variant was included based on its report as a VUS in ClinVar. Additional information was not available. |
| c.501A>C p.(Lys167Asn) | c.582A>C p.(Lys194Asn) | g.34859505T>G | CA410207998 | RCV000680423.1 | – | 0.826 | DiNardo et al. reported this variant in a 46-year-old male with thrombocytopenia and his daughter being diagnosed with MDS with del(5q) at the age of 5 years [9]. |
| c.538C>T p.(Arg180Trp) | c.619C>T p.(Arg207Trp) | g.34834596G>A | CA410207207 | RCV000503595.1 | – | 0.811 | The variant was included based on its report as a VUS in ClinVar. Additional information was not available. |
| c.614G>A p.(Arg205Gln) | c. 695G>A p.(Arg232Gln) | g.34834520C>T | CA10014376 | – | rs368711448 | 0.583 | The variant was reported in a child with refractory cytopenia in childhood that underwent HSCT. Subsequently, the variant was identified in her healthy mother and a healthy sisters. |

**C Classification of variants of interest**

| **RUNX1b (NM_001001890.3)** | **RUNX1c (NM_001754.5)** | **Chr21 (GRCh38)** | **Variant classification** | | | |
| --- | --- | --- | --- | --- | --- | --- |
|  |  |  | **criteria w/o functional data** | **prelim. class** | **functional criteria** | **classification** |
| c.86T>C p.(Leu29Ser) **^#^** | c.167T>C p.(Leu56Ser) | g.34887027A>G | BA1, BP2 | 1 | uncertain function | 1 |
| c.178G>T p.(Gly60Cys) | c.259G>T p.(Gly87Cys) | g.34886935C>A | PP3 | 3 | functional (BS3_strong) | 3 |
| c.235T>A p.(Trp79Arg) **^#^** | c.316T>A p.(Trp106Arg) | g.34886878A>T | PM1_supporting, PM2_moderate, PP3_supporting | 3 ^§^ | non-functional (PS3_strong) | 4 |
| c.350T>A p.(Leu117Gln) | c.431A>C p.(Leu144Gln) | g.34880634A>T | PS4_supporting, PM2_moderate, PM1_supporting, PP3_supporting | 3 | likely non-functional (PS3_moderate) | 4 |
| c.352A>G p.(Arg118Gly) | c.433A>G p.(Arg145Gly) | g.34880632T>C | PS4_supporting, PM1_supporting, PM2_moderate, PP3_supporting | 3 | likely non-functional (PS3_moderate) | 4 |
| c.473A>C p.(Gln158Pro) | c.554A>C p.(Gln185Pro) | g.34859533T>G | PM1_supporting, PM2_moderate, PP3_supporting | 3 | non-functional (PS3_strong) | 4 |
| c.501A>C p.(Lys167Asn) | c.582A>C p.(Lys194Asn) | g.34859505T>G | PS4_supporting, PM1_supporting, PM2_moderate, PP3_supporting | 3 | non-functional (PS3_strong) | 4 |
| c.538C>T p.(Arg180Trp) | c.619C>T p.(Arg207Trp) | g.34834596G>A | PM2_moderate, PP3_supporting | 3 | uncertain function | 3 |
| c.614G>A p.(Arg205Gln) | c. 695G>A p.(Arg232Gln) | g.34834520C>T | PS4_supporting | 3 | likely functional (BS3_supporting) | 3 |

**Legend of 2A-C:** Variant nomenclature is given in accordance to HGVS recommendations. Additionally, we listed the genomic localization (GRCh38) and, if available, entries in ClinVar, dbSNP, REVEL score [10], and clinical data.

**#,** Variants marked with this symbol had been classified as variants of uncertain significance (VUS) when integrated in the present study. However, they have been reclassified while performing the present investigation based on the MM‑VCEP recommendations;

**§,** Please note that based on the functional data of Tsai and colleagues [11], this variant has been classified as class 4 by the MM-VCEP [7] while performing the present study. As discussed in the main text, our data confirms this classification and the functional data previously reported;

**MDS-EB**, MDS with excess blasts;

**HSCT**, Hematopoietic stem cell transplantation;

**MUD**, Matched unrelated donor;

**MDR-AML**, MDS-related AML;

**MM-VCEP**, ClinGen Myeloid Malignancy Variant Curation Expert Panel [7].

**3. Supplementary Figures and Table**

**
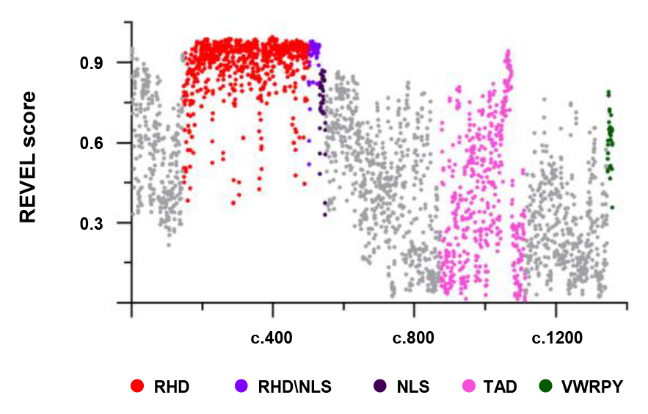
**

**Supplementary Fig. 1 Precalculated REVEL scores.** REVEL scores [10] of all RUNX1 missense variants are plotted with respect to their cDNA position. RUNX1 protein domains are color-coded (red, RHD; light violet, RHD\nuclear localization signal (NLS) domain; dark violet, NLS; pink, transactivation domain (TAD); green, VWRPY domain (VWRPY)).


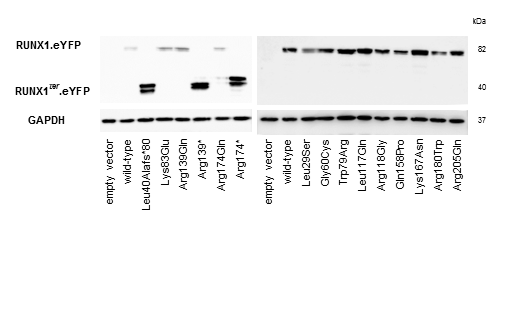


**Supplementary Fig. 2 Representative Western Blot analyzing HEK293T transfection conditions used for FRET assay.** HEK293T cells were cotransfected with eYFP and eCFP fusion expression plasmids for RUNX1(variants) and CBFB.


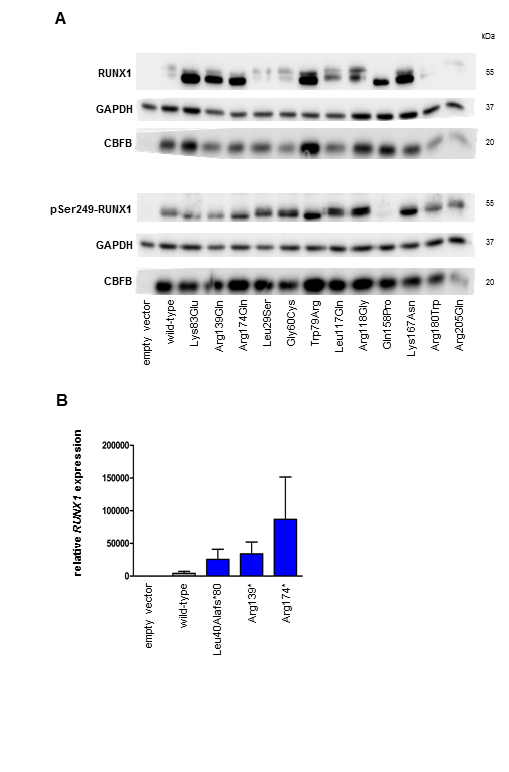

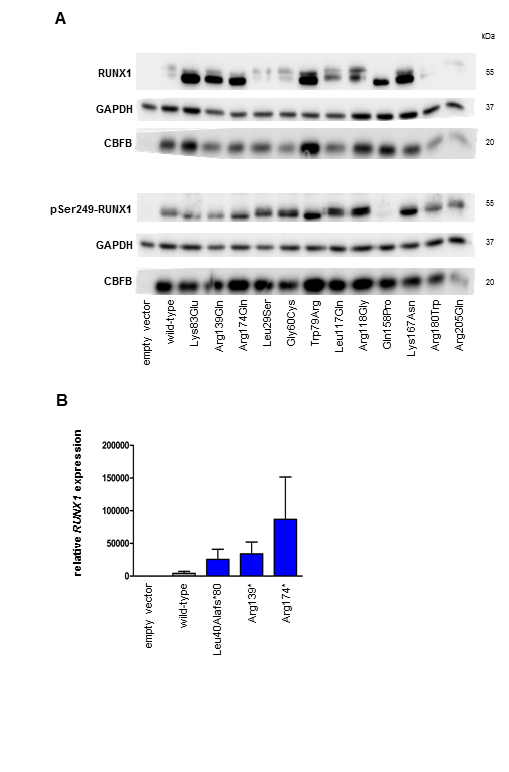


**Supplementary Fig. 3 Representative Western Blot (a) and qRT-PCR (b) analyzing HEK293T transfection conditions used for luciferase reporter assays and quantification of RUNX1 phosphorylation.** HEK293Tcells were cotransfected with pcDNA3.1 expression plasmids for RUNX1(variants) and CBFB.


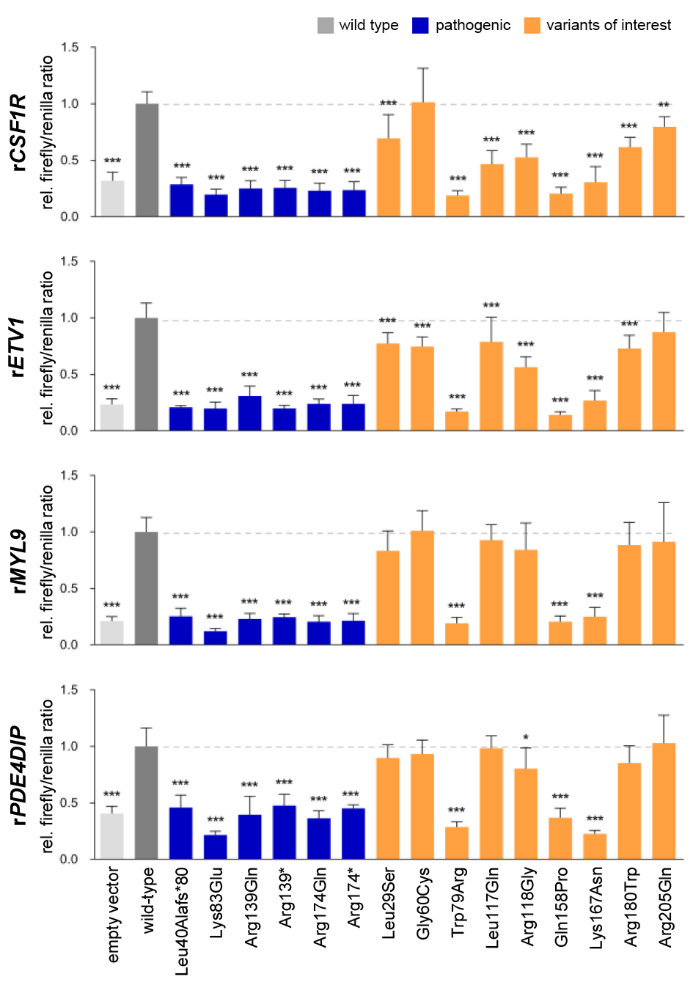


**Supplementary Fig. 4 Transcriptional activation of luciferase reporter constructs in HEK293T cells.** The bar graphs display from top to bottom the firefly/renilla ratios relative to wild-type RUNX1 for the reporter constructs r*CSF1R*, r*ETV1*, r*MYL9*, and r*PDE4DIP* (mean+SD; 3 biological and 3 technical replicates; one-way ANOVA; Dunnett’s post hoc test; *, *P* ≤.05; **, *P* ≤.01; ***, *P* ≤.001).


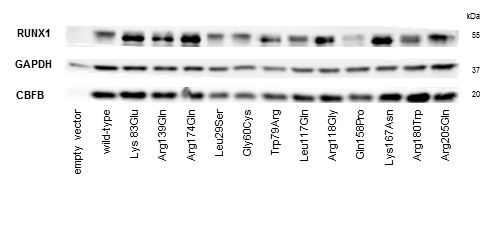


**Supplementary Fig. 5. Representative Western Blot analyzing HEL transfection conditions used for luciferase reporter assays.** HEL cells were cotransfected with pcDNA3.1 expression plasmids for RUNX1(variants) and CBFB.


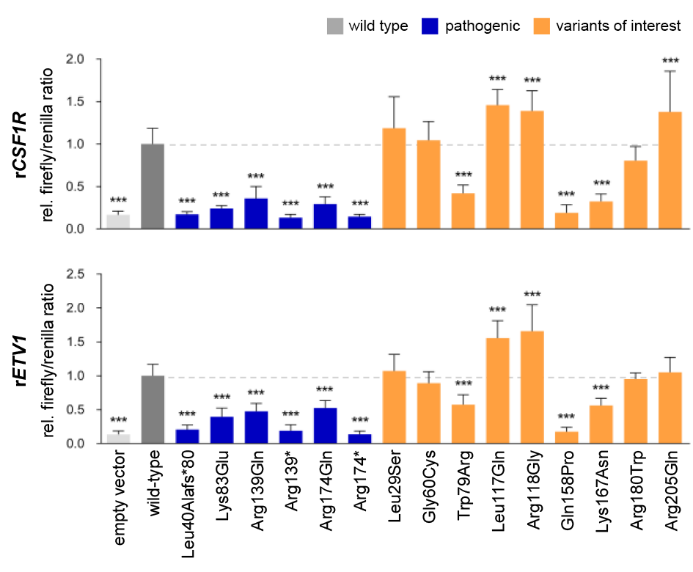


**Supplementary Fig. 6 Transcriptional activation of luciferase reporter constructs in HEL cells.**

The top and lower bar graphs display the firefly/renilla ratios relative to wild-type RUNX1 for the reporter constructs r*CSF1R* and r*ETV1*, respectively (mean+SD; 2 biological and 5 technical replicates; one-way ANOVA; Dunnett’s post hoc test; *, *P* ≤.05; **, *P* ≤.01; ***, *P* ≤.001).

**Supplementary Table 1. Primer sequences for cloning and site directed mutagenesis (SDM).**

| **oligo name** | **sequence** | **purpose** |
| --- | --- | --- |
| CBFB_*Hind*III_f | GGCCAAGCTTGCCACCATGCCGCGCGTCGTGCCC | CBFB cDNA amplification |
| CBFB_*Xho*I_r | CGCGTCTCGAGCTAGGGTCTTGTTGTCTTCTTGCCAG |  |
| RUNX1_*EcoR*I_f | CATGGAATTCGCCACCATGCGTATCCCCGTAGATG | RUNX1 cDNA amplification |
| RUNX1_*Not*I_r | GAAATGCGGCCGCTCAGTAGGGCCTCCACACGG |  |
|  |  |  |
| RUNX1_C1c_*Nhe*I_f | CAACGCTAGCATGCGTATCCCCGTAGATGCCAGCACG | expression plasmids FRET |
| RUNX1_C1c_*Age*I_r | GATTACCGGTGCACCTGCTCCGTAGGGCCTCCACACGG |  |
| CBFB_C1c_*Nhe*I_f | ATTAGCTAGCATGCCGCGCGTCGTGCCCGACCAGAGAAG |  |
| CBFB_C1c_*Age*I_r | GCCAACCGGTGCACCTGCTCCGGGTCTTGTTGTCTTCTTG |  |
|  |  |  |
| RUNX1_C520T_fw | CAGTGGATGGGCCCTGAGAACCTCGAAGA | side directed mutagenesis |
| RUNX1_C520T_rev | TCTTCGAGGTTCTCAGGGCCCATCCACTG |  |
| RUNX1_T350A_fw | ACTACTCGGCTGAGCAGAGAAATGCTACCGC |  |
| RUNX1_T350A_rev | GCGGTAGCATTTCTCTGCTCAGCCGAGTAGT |  |
| RUNX1_G178T_fw | CCGACCACCCGTGCGAGCTGGTG |  |
| RUNX1_G178T_rev | CACCAGCTCGCACGGGTGGTCGG |  |
| RUNX1_*Not*I_r | GAAATGCGGCCGCTCAGTAGGGCCTCCACACGG |  |
| RUNX1_dup90_117_*Mre*I | ATTACGCCGGCGCTGCCGCTGGGCGCCCCGGACGCCG |  |
| RUNX1_sdm614neu_f | CTGGAGCAGCTGCAGCGCACAGCCATG |  |
| RUNX1_sdm614neu_r | CATGGCTGTGCGCTGCAGCTGCTCCAG |  |
| RUNX1_sdm235_f | GTGCTGCCTACGCACAGGCGCTGCAACAAG |  |
| RUNX1_sdm235_r | CTTGTTGCAGCGCCTGTGCGTAGGCAGCAC |  |
| RUNX1_sdm352_f | CTCGGCTGAGCTGGGAAATGCTACCGC |  |
| RUNX1_sdm352_r | GCGGTAGCATTTCCCAGCTCAGCCGAG |  |
| RUNX1_sdm538_f | CGAGAACCTCGAAGACATTGGCAGAAACTAGATGATC |  |
| RUNX1_sdm538_r | GATCATCTAGTTTCTGCCAATGTCTTCGAGGTTCTCG |  |
| RUNX1_sdm415_f | CCTCAGGTTTGTCGGTTGAAGTGGAAGAGGGAA |  |
| RUNX1_sdm415_r | TTCCCTCTTCCACTTCAACCGACAAACCTGAGG |  |
| RUNX1_sdm247_f | CACTGGCGCTGCAACGAGACCCTGCCCATCG |  |
| RUNX1_sdm247_r | CGATGGGCAGGGTCTCGTTGCAGCGCCAGTG |  |
| RUNX1_sdm416_f | CCTCAGGTTTGTCGGTCAAAGTGGAAGAGGGAA |  |
| RUNX1_sdm416_r | TTCCCTCTTCCACTTTGACCGACAAACCTGAGG |  |
| RUNX1_sdm521_f | CAGTGGATGGGCCCCAAGAACCTCGAAGACA |  |
| RUNX1_sdm521_r | TGTCTTCGAGGTTCTTGGGGCCCATCCACTG |  |
| RUNX1_sdm86_f | AGATGAGCGAGGCGTcGCCGCTGGGCGCCCC |  |
| RUNX1_sdm86_r | GGGGCGCCCAGCGGCGACGCCTCGCTCATCT |  |
| RUNX1_sdm473_f | TCACAAACCCACCGCCAGTCGCCACCTACCA |  |
| RUNX1_sdm473_r | TGGTAGGTGGCGACTGGCGGTGGGTTTGTGA |  |
|  |  |  |
| rCSFR1_*Sac*I_short_fw | GTACGAGCTCAGATATGCATTACTTTGGAGATTC | luciferase reporter |
| rCSF1R_*Sac*I_r | GGCACAGAGCTCTCAGCTAC |  |
| rETV1_*Xho*I_f | CATGCTCGAGTCTAAGGGTATACCAGAACC |  |
| rETV1_*Hind*III_r | CATGAAGCTTGCTGCTGCTCTTCGCAAATC |  |
| rMYL9_*Xho*I_fw | AAAACTCGAGGGGCAGCAGAGGGATGGAG |  |
| rMYL9_*Hind*III_rev | GGTTAAGCTTACATCTTGGCTTCTGGTGGG |  |
| rPDE4DIP.*Xho*I_F | CATGCTCGAGGATAGGGCTGGGGTTAAAAC |  |
| rPDE4DIP.*Hind*III_R | CATGAAGCTTGGATTTCCTGGGCCTGGCC |  |

**References – Supplemental data**

1. Banning C, Votteler J, Hoffmann D, Koppensteiner H, Warmer M, Reimer R, et al. A flow cytometry-based FRET assay to identify and analyse protein-protein interactions in living cells. PLoS One. 2010;5:e9344.

2. Jalagadugula G, Mao G, Kaur G, Goldfinger LE, Dhanasekaran DN, Rao AK. Regulation of platelet myosin light chain (MYL9) by RUNX1: Implications for thrombocytopenia and platelet dysfunction in RUNX1 haplodeficiency. Blood. 2010;116:6037–45.

3. Zhang DE, Hetherington CJ, Chen HM, Tenen DG. The macrophage transcription factor PU.1 directs tissue-specific expression of the macrophage colony-stimulating factor receptor. Mol Cell Biol. 1994;14:373–81.

4. Michaud J, Wu F, Osato M, Cottles GM, Yanagida M, Asou N, et al. In vitro analyses of known and novel RUNX1/AML1 mutations in dominant familial platelet disorder with predisposition to acute myelogenous leukemia: Implications for mechanisms of pathogenesis. Blood. 2002;99:1364–72.

5. Song WJ, Sullivan MG, Legare RD, Hutchings S, Tan X, Kufrin D, et al. Haploinsufficiency of CBFA2 causes familial thrombocytopenia with propensity to develop acute myelogenous leukaemia. Nat Genet. 1999;23:166–75.

6. Ripperger T, Tauscher M, Haase D, Griesinger F, Schlegelberger B, Steinemann D. Managing individuals with propensity to myeloid malignancies due to germline RUNX1 deficiency. Haematologica. 2011;96.

7. Luo X, Feurstein S, Mohan S, Porter CC, Jackson SA, Keel S, et al. ClinGen Myeloid Malignancy Variant Curation Expert Panel recommendations for germline RUNX1 variants. Blood Adv. 2019;3:2962–79.

8. Owen CJ, Toze CL, Koochin A, Forrest DL, Smith CA, Stevens JM, et al. Five new pedigrees with inherited RUNX1 mutations causing familial platelet disorder with propensity to myeloid malignancy. Blood. 2008;112:4639–45.

9. DiNardo CD, Bannon SA, Routbort M, Franklin A, Mork M, Armanios M, et al. Evaluation of Patients and Families With Concern for Predispositions to Hematologic Malignancies Within the Hereditary Hematologic Malignancy Clinic (HHMC). Clin Lymphoma Myeloma Leuk. 2016;16:417–28.

10. Ioannidis NM, Rothstein JH, Pejaver V, Middha S, McDonnell SK, Baheti S, et al. REVEL: An Ensemble Method for Predicting the Pathogenicity of Rare Missense Variants. Am J Hum Genet. 2016;99:877–85.

11. Tsai SC, Shih LY, Liang ST, Huang YJ, Kuo MC, Huang CF, et al. Biological activities of RUNX1 mutants predict secondary acute leukemia transformation from chronic myelomonocytic leukemia and myelodysplastic syndromes. Clin Cancer Res. 2015;21:3541–51.
